# Sero-molecular prevalence of toxoplasmosis in hemodialysis and peritoneal dialysis patients in Markazi Province, Iran

**DOI:** 10.1186/s41182-024-00676-3

**Published:** 2025-01-14

**Authors:** Hossein Sarmadian, Mana Shojapour, Fereshteh chegeni, Mohammad Amin Tabatabaiefar, Farshid Haghverdi, Roham Sarmadian, Reza Ghasemikhah

**Affiliations:** 1 Department of Infectious Disease, School of Medicine, Arak University of Medical Sciences, Arak, Iran; 2 Molecular and Medicine Research Center, Arak University of Medical Sciences, Arak, Iran; 3 Students Research Committee, Arak University of Medical Sciences, Arak, Iran; 4 Department of Genetics and Molecular Biology, School of Medicine, Isfahan University of Medical Sciences, Isfahan, Iran; 5 Nephrology Department of Internal Medicine, School of Medicine, Amiralmomenin Hospital Arak University of Medical Sciences, Arak, Iran; 6 Infectious Diseases Research Center, Arak University of Medical Sciences, Arak, Iran; 7 Department of Parasitology and Mycology, School of Medicine, Arak University of Medical Sciences, Arak, Iran

**Keywords:** Toxoplasmosis, Hemodialysis, ELISA, PCR

## Abstract

**Background:**

Infectious diseases, particularly parasitic infections such as toxoplasmosis, contribute significantly to the morbidity and mortality of hemodialysis patients. *Toxoplasma gondii* infection poses serious risks, especially to immunocompromised individuals. This study aimed to assess the prevalence of latent toxoplasmosis in dialysis patients in Markazi Province, Iran.

**Methods:**

A total of 181 patients (168 hemodialysis and 13 peritoneal dialysis) were included in this cross-sectional study. Blood samples were collected and tested for anti-Toxoplasma IgG and IgM antibodies using ELISA, while PCR was used to detect *T. gondii* DNA. Data were analyzed using SPSS, with P < 0.05 considered significant.

**Results:**

Out of 181 patients, 123 (67.95%) were seropositive for IgG antibodies, with the highest prevalence in those aged over 60 years. No cases tested positive for IgM antibodies or *T. gondii* DNA. Age was significantly associated with higher seroprevalence (P < 0.05), but there were no significant differences regarding gender, dialysis type, or duration of dialysis.

**Conclusion:**

The high prevalence of latent toxoplasmosis in dialysis patients underscores the need for regular screening to prevent serious complications. Given the limitations of serological tests, molecular diagnostics like PCR should be considered for better detection in immunocompromised patients.

## Background

*Toxoplasma gondii* (*T. gondii*) is a widely distributed protozoan parasite that infects both humans and animals. It is estimated that around 30% of the global human population is infected, and in immunocompromised individuals, fetuses, and newborns, it can lead to severe and potentially life-threatening diseases [1, 2]. *T. gondii* has three primary genotypes—type I, type II, and type III—that differ in their disease-causing potential and prevalence in humans [3–5].

Members of the Felidae family, such as domestic cats, serve as the definitive hosts of *T. gondii*, with the parasite’s sexual cycle taking place in the intestinal epithelium of these animals. In intermediate hosts, the parasite exists in two forms: tachyzoites and bradyzoites. Bradyzoites, which are tissue cysts found in the brain and muscles, are responsible for chronic infection. When the host's immune system is weakened, these bradyzoites convert into tachyzoites, the active form of the parasite, leading to acute infection [1, 6, 7].

*T. gondii* can be transmitted through various routes, including ingestion of oocysts from contaminated food or water, consumption of undercooked meat containing tissue cysts, vertical transmission from mother to fetus, organ transplantation, or blood transfusion [6, 8].

Acute toxoplasmosis is often asymptomatic and self-limiting; however, individuals with weakened immune systems, such as cancer patients undergoing chemotherapy, AIDS patients, those with thalassemia receiving multiple transfusions, transplant recipients, and hemodialysis patients, are at the highest risk of developing severe toxoplasmosis [9].

Toxoplasmosis is a significant concern for patients with chronic kidney disease, particularly those undergoing hemodialysis who are vulnerable due to their compromised immune systems [10].

The morbidity and mortality associated with this infection necessitate effective diagnostic methods to identify and manage affected patients. Toxoplasmosis is typically diagnosed by detecting specific IgG and IgM antibodies against Toxoplasma. However, these methods have limitations, especially in immunocompromised patients. Recent advancements in diagnostic procedures for *Toxoplasma gondii*, particularly polymerase chain reaction (PCR), have greatly enhanced detection capabilities. PCR allows for directly identifying *T. gondii* DNA in blood samples, improving sensitivity and enabling the diagnosis of active infections [11]. This study aims to assess toxoplasmosis in individuals undergoing peritoneal dialysis and hemodialysis recipients.

## Material and methods

### Study population and sample collection

This cross-sectional study involved hemodialysis and peritoneal dialysis patients from Markazi Province, Iran. All participants provided written informed consent after being informed of the study’s purpose. Ethical approval was obtained from the Institutional Human Ethics Committee of Arak University of Medical Sciences (Approval No. IR.ARAKMU.REC.1396, 256). A 5-mL blood sample was collected from each participant, which was then divided into aliquots for ELISA and molecular tests. The samples were sent to the laboratory at the Arak Faculty of Medical Sciences. The serum was separated by centrifugation at 3000 rpm (1500 × g) for 15 min and stored at − 20 °C for ELISA testing.

### Enzyme-linked immunosorbent assay (ELISA)

Serum samples were analyzed for specific Toxoplasma antibodies using a commercial Anti-Toxoplasma IgG & IgM ELISA kit (ACON, San Diego, CA, USA), following the manufacturer’s instructions.

### DNA extraction and polymerase chain reaction (PCR)

Genomic DNA was extracted from whole blood using the DNA extraction kit (YektatajhizAzma, Iran) according to the manufacturer’s guidelines and stored at − 20 °C until analysis. PCR amplification of the B1 gene was performed using B1 forward (TCGCAGTACACCAGGAGTTG) and B1 reverse (CTCCGCAGCGACTTCTATCT) primers. The PCR reaction was conducted in a 25 µl volume, which included 12.5 µl of master mix, 8.5 µl of sterile distilled water, 2 µl of template DNA, and 1 µl of each primer. A positive control (strain containing the target gene) and a negative control (nuclease-free water) were included. The thermal cycling conditions consisted of an initial denaturation at 94 °C for 4 min, followed by 35 cycles of denaturation at 94 °C for 30 s, annealing at 55 °C for 30 s, extension at 72 °C for 30 s, and a final extension at 72 °C for 5 min. The amplified PCR products (288 bp) were analyzed by electrophoresis on a 2% agarose gel and visualized using a transilluminator.

### Data analysis

Data were analyzed using SPSS software (version 27), with a significance level set at P < 0.05.

## Results

This study was conducted on 181 individuals, including 168 hemodialysis patients and 13 peritoneal dialysis patients in Markazi Province. Among the participants, 95(52.5%) were male and 86(47.5%) were female.

**Fig. 1:**
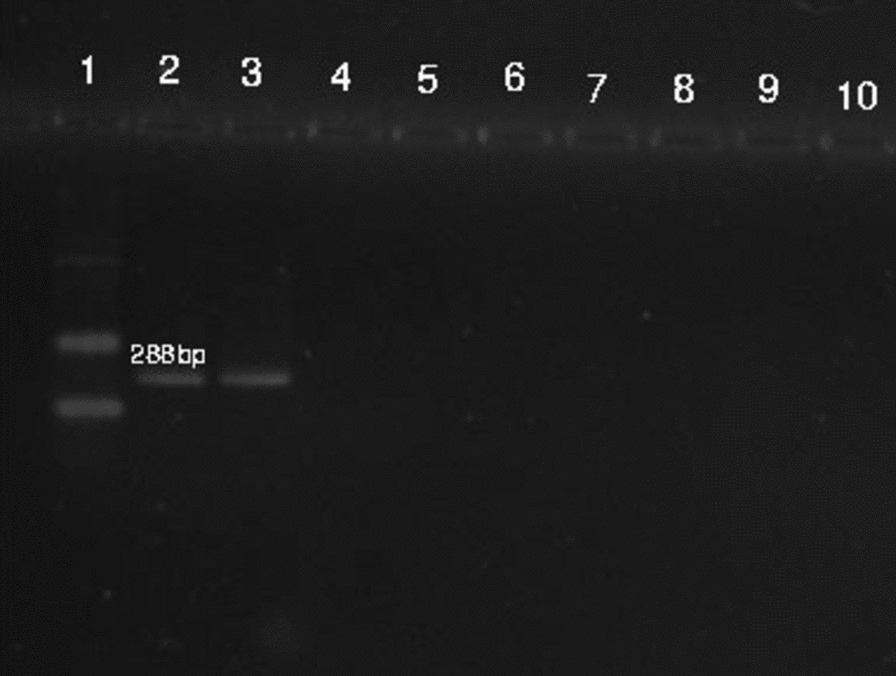
Agarose gel electrophoresis (2% agarose) of PCR amplified products. Lane 1: (ladder 100 bp), lanes 2, 3: positive control (288 bp), lane 4: negative control, lanes 5–10: negative samples

The results showed that 123(67.95%) individuals had IgG antibodies against *Toxoplasma gondii*, and none of the patients tested positive for IgM antibodies or PCR (Fig. 1).

The highest prevalence was observed in the age group of > 60 years, and statistical analysis indicated a significant relationship between increasing age and the incidence of the infection. Additionally, the frequency of IgG antibodies in females was higher than in males, but there was no significant difference between gender and the prevalence of this protozoan (P < 0.05).

Furthermore, our finding showed that no significant difference was observed between the seropositivity of *Toxoplasma gondii* in hemodialysis patients and peritoneal dialysis patients (P < 0.05). Also, this study found no significant relationship between the serum prevalence of IgG antibodies against Toxoplasma in hemodialysis patients and the duration of their dialysis (P < 0.05) (Table 1).

**Table 1:** Sero-molecular prevalence of *Toxoplasma gondii* infection in different groups

Characteristic	Anti-*T. gondii* IgG seropositivity (number/ percent)	Anti-*T. gondii* IgM seropositivity (number/ percent)	PCR	*P*-value
Age group (years)				
< 20	1(0.5%)	0	0	0.01
21–40	11(6%)	0	0	
41–60	45(24.5%)	0	0	
> 60	66(35.8%)	0	0	
Sex				
Female	60(69%)	0	0	0.33
Male	63(64.9%)	0	0	
Procedure				
Hemodialysis	113(67.3%)	0	0	0.13
Peritoneal dialysis	9(69.2%)	0	0	
Duration of dialysis				
Low 1 year	76%	0	0	0.66
1–5 years	71.9%	0	0	
Up 5 years	77.1%	0	0	

## Discussion

Infectious complications remain a leading cause of increased morbidity and mortality in patients undergoing hemodialysis, with parasitic infections, such as blastocystosis, cryptosporidiosis, and toxoplasmosis, being particularly concerning due to their high prevalence. Toxoplasmosis, in particular, can lead to severe complications in dialysis patients, sometimes resulting in death, thereby imposing significant burdens on healthcare systems. A national meta-analysis conducted in 2018, which reviewed 10 studies, found a positive correlation between *Toxoplasma gondii* exposure and hemodialysis [12].

In the present study, the prevalence of anti-*Toxoplasma gondii* IgG antibodies was found to be 67.95% among the total patients, indicating a considerable rate of infection. Several epidemiological studies have been conducted in different regions of Iran and other countries, but none have focused specifically on the seroepidemiology of *T. gondii* among dialysis patients in Markazi Province. Previous research on the general population revealed a 33.5% seroprevalence of anti-*Toxoplasma* antibodies (276 out of 825 individuals) [13]. Similarly, a study by Kadkhodaei et al. (2023) found an anti-*T. gondii* IgG prevalence of 18.66% in hemodialysis patients from Kazeroon and 25.33% from Jahrom [14]. Other studies have reported varying prevalence rates in different parts of Iran, such as Khuzestan (49.5%) [15], Bushehr (40%) [16], Sari (80.8%) [17], Kashan and Qom (63%) [10], Sistan and Baluchestan (73.7%) [18], Tehran (67.3%) [19], Yasuj (30%) [20], and Guilan (74%) [21].

Internationally, studies have also shown different prevalence rates of toxoplasmosis in hemodialysis patients, including 41.7% in Senegal [22], 54.1% in Iraq [23], 59.3% in Turkey [24], and 60% in Egypt[25]. These differences in prevalence rates across regions are likely due to factors such as climate, living conditions, hygiene practices, soil exposure, education, occupation, dietary habits, socioeconomic status, and pet ownership, particularly the keeping of cats.

In our study, we observed that the highest prevalence of infection was found in the age group of > 60 years, with statistical analysis indicating a significant relationship between increasing age and the incidence of toxoplasmosis. This finding aligns with previous research suggesting that older adults may be more susceptible to infections due to a combination of risk factors, including increased exposure to toxoplasma over time, changes in immune response with aging, and comorbidities [26]. The results of our study indicated that there was no significant association between gender and Toxoplasma infection. Additionally, the findings of other similar studies were consistent with our results [27, 28].

Serological testing for IgG and IgM antibodies is a common method for diagnosing toxoplasmosis, though it has certain limitations. For instance, IgM antibodies can persist for extended periods and may be detectable during chronic infection, increasing the risk of false positives due to cross-reactivity with other antibodies or diseases. Additionally, specific antibodies may not appear during the early stages of infection, especially in immunocompromised patients. Moreover, changes in antibody levels during Toxoplasma reactivation are not always apparent, making serological diagnosis of active infection unreliable. As a result, molecular methods such as PCR are increasingly recognized as valuable diagnostic tools, particularly for detecting toxoplasmosis in immunocompromised patients [29, 30].

The present study found no positive cases for IgM or *T. gondii* DNA, consistent with the findings of Kadkhodaei et al., who reported no detection of IgM or *T. gondii* DNA in either the case or control groups [14]. Similarly, the study by Hamidi et al. on hemodialysis and peritoneal dialysis patients found no serological evidence of IgM antibodies [28].

In contrast, Yarahmadi et al. detected *T. gondii* DNA in 29.55% of blood samples using nested PCR [31], and a study by Nahnoush et al. reported IgM seropositivity and *Toxoplasma* DNA in 14.6% and 2% of hemodialysis patients, respectively [25]. In a study by Rezavand et al.,54 (60%) of hemodialysis patients tested positive for anti-Toxoplasma IgG, 3(3.3%) for IgM, and 5(6%) for *T. gondii* DNA using PCR)among 5 samples, all tested positive for IgG, while only 3 tested positive for IgM). The results showed that the sensitivity and specificity of PCR in toxoplasma diagnosis were 100% and 98.9%, respectively [32]. In the 2024 study by El-Askary, 61% of patients were IgG positive and 22.7% IgM positive. Among these cases, only one case was reported with a positive PCR test (IgM negative and IgG positive) [33]. The researchers noted that in toxoplasma reactivation, or early stages of infection, antibody levels are not reliable. Also, in cases where IgM was positive but molecular testing was negative, this could indicate persistent IgM levels in chronic infection or a false-positive serological test result. Furthermore, in our study, considering the negative PCR results in all samples, the parasite load in the blood may have been low. Therefore, it is advisable to use molecular tests with higher sensitivity, such as real-time PCR.

Our study found no significant difference in *Toxoplasma* seroprevalence between hemodialysis and peritoneal dialysis patients, and the duration of dialysis treatment did not influence the seropositivity rate. These findings are consistent with previous research [17, 28, 34].

Overall, this study demonstrated a high prevalence of *T. gondii* infection among dialysis patients in this region and highlighted toxoplasmosis as a serious risk factor for this population. We recommend regular screening for *T. gondii* infection as part of routine care for dialysis patients to prevent transmission to others during dialysis and reduce mortality. In Iran, routine screening for individuals with Toxoplasma includes serological tests (IgM and IgG). However, in dialysis patients, due to their weakened immune systems and increased risk of infection, choosing appropriate methods for screening for Toxoplasma infection is particularly important. In this context, combining serological and highly sensitive molecular tests, such as real-time PCR, typically yields the best results. If suspicious results are observed, follow-up and repeat testing are recommended to ensure accurate diagnosis and appropriate management.

## Data Availability

No datasets were generated or analysed during the current study.
